# External validation of the Surgical Outcome Risk Tool (SORT) in 3305 abdominal surgery patients in the independent sector in the UK

**DOI:** 10.1186/s13741-020-00173-1

**Published:** 2021-01-26

**Authors:** K. Oakland, D. Cosentino, T. Cross, C. Bucknall, S. Dorudi, D. Walker

**Affiliations:** 1 Digestive Diseases and Renal Department, HCA Healthcare UK, 242 Marylebone Road, London, NW1 6JL UK; 2 Clinical Informatics Department, HCA Healthcare UK, 242 Marylebone Road, London, NW1 6JL UK; 3 Princess Grace Hospital, HCA Healthcare UK, 42-52 Nottingham Place, London, W1U 5NY UK; 4 Centre for Perioperative Medicine, University College London, Gower Street, London, WC1E 6BT UK

**Keywords:** Pre-assessment, Surgery, Risk assessment, Outcomes

## Abstract

**Background:**

Assessing the risk of post-surgical mortality is a key component of pre-surgical planning. The Surgical Outcome Risk Tool (SORT) uses pre-operative variables to predict 30-day mortality. The aim of this study was to externally validate SORT in patients undergoing major abdominal surgery.

**Methods:**

Data were collected from patients treated in five independent hospitals in the UK. Individualised SORT scores were calculated, and area under the receiver operating characteristic (AUROC) and precision-recall curves (PRC) plus 95% confidence intervals (CI) were drawn to test the ability of SORT to identify in-hospital death. Outcomes of patients with a SORT predicted risk of mortality of ≥ 5% (high risk) were compared to those with a predicted risk of < 5% (standard risk).

**Results:**

The study population comprised 3305 patients, mean age 51 years, 2783 (84.2%) underwent elective surgery most frequently involving the colon (24.6%), or liver, pancreas or gallbladder (18.2%). Overall, 1551 (46.9%) patients were admitted to ICU and 29 (0.88%) died. The AUROC of SORT for discriminating patients at risk of death in hospital was 0.899 (95% CI 0.849 to 0.949) and the PRC 0.247. In total, 72 (2.18%) patients were stratified as high risk. There were more unplanned ICU admissions and deaths in this group compared to the standard risk group (25.0% and 3.3%, versus 3.1% and 0.5%, respectively).

**Conclusion:**

We externally validated SORT in a large population of abdominal surgery patients. SORT performed well in patients with lower risk profiles, but underpredicted adverse outcomes in the higher risk group.

## Introduction

In 2010, the National Confidential Enquiry into Patient Outcome and Death (NCEPOD) conducted a national review of care provided to high-risk surgical patients. A key finding was the need for a UK-wide system that could reliably identify patients at high risk of mortality and morbidity (National Confidential Enquiry into Patient Outcomes and Death, 2011). In 2018, the Royal College of Surgeons of England (RCS) formalised this, recommending that all adult patients admitted under the care of a general surgeon should have their risk of morbidity and mortality assessed and recorded. The RCS also recommended that high-risk surgical patients, defined as those with a predicted mortality of ≥ 5%, should receive timely surgery in the presence of a consultant surgeon and should immediately be admitted to critical care post-operatively (Royal College of Surgeons of England, 2018). Prediction tools have been developed to quantify risk of death or morbidity, but have either not been designed to generate individualised risk profiles or require variables that are only available intra-operatively, limiting their use in the pre-operative setting.

Recently, the Surgical Outcome Risk Tool (SORT) was developed from a population of 16,788 patients who underwent elective or emergency general, head and neck, ophthalmology, orthopaedic, thoracic, urology or vascular surgery with the aim of predicting 30-day mortality following surgery (Protopapa et al., 2014) but as yet has not been fully externally validated. It comprises procedure code, operation severity, American Society of Anaesthesiologists’ physical status classification (ASA), clinical urgency, surgical site (thoracic, gastrointestinal or vascular surgery), cancer (active malignancy within the last 5 years) and age, all of which are available pre-operatively.

The aims of this study were to (1) externally validate the ability of SORT to predict in-hospital mortality and (2) to assess its use in determining elective ICU admission in a large population of general surgical patients admitted to and treated in five independent hospitals in the United Kingdom (UK).'

## Methods

This study was conducted across five independent hospitals, operated by HCA Healthcare UK in London. All participating hospitals had a 24/7 level 3 intensive care unit (ICU) and on site access to interventional radiology and emergency theatres.

### Patient population

We studied all insured adult patients who underwent elective and emergency major abdominal surgery in a HCA facility between 1 January 2013 and 30 September 2018. Major abdominal surgery was defined using the Clinical Coding and Schedule Development Group (CCSD) schedule of procedures, comprising 125 individual procedures within the following groups of codes: stomach, duodenum, small intestine, large intestine, rectum, repair of a major vessel, oesophagus, other abdominal organs and peritoneum (Appendix 1). Patients with procedures classified as minor or intermediate were excluded. If a patient had multiple procedures performed synchronously (with separate procedure codes), the most complex procedure code was used to calculate SORT. Procedure codes that were associated with a discrete hospital admission were considered as separate cases.

Patients who were transferred to other hospitals (National Health Service, NHS, or other independent hospitals beyond HCA) were excluded from the analyses as it was not possible to collect data on their clinical outcomes after transfer. It was also not possible to determine why these patients were transferred to the NHS. Routine administrative data that are collected prospectively on patient demographics, surgical procedure, ASA and patient outcomes were used for this study. These data are collected automatically or by clinical or administrative staff and are entered directly into hospitals’ electronic health records. Post-operative ICU admission defined as level 2 or 3 care and was classified as planned or unplanned. These data were entered into the electronic health record by clinical staff at the point of admission to ICU. However for some patients, it was not clear from the data whether the reason for ICU admission was due to clinical need or lack of ward capacity. These cases were handled as missing data in the analysis. Post-surgery ICU admission was limited to ICU admissions that occurred within 7 days of surgery. In cases of multiple ITU admissions during the same hospital episode of care, only the first admission after surgery was considered.

The study proposal was reviewed and approved by the hospitals’ Research Review Committee who deemed that ethical approval was not required as no new data were collected, and the study involved no patient intervention. The study was performed and reported in accordance with the TRIPOD statement (Collins et al., 2015).

### Applying SORT

SORT was calculated for each patient. SORT classifies the ‘procedure urgency’ variable using the NCEPOD classification of interventions: immediate (within minutes of decision to operate), urgent (within hours), expedited (within days) or elective (routine admission) (National Confidential Enquiry into Patient Outcomes and Death, 2015). However, the hospitals’ electronic database defined this variable as only ‘elective’ or ‘unplanned’. Due to the nature of surgical cases in the Independent sector, true ‘immediate’ cases would be extremely rare. It was not possible to differentiate ‘urgent’ from ‘expedited’ so these variables were grouped together.

### Study outcomes

The primary outcome of interest was all-cause in-hospital mortality. We used in-hospital death as opposed to 30-day mortality as it was not possible to collect outcome data after hospital discharge. The RCS defines ‘high-risk’ patients as those with a risk of death of ≥ 5% (Royal College of Surgeons of England, 2018). The applicability of SORT-generated mortality predictions was tested by using each patients’ predicted risk to stratify need for ICU admission. SORT-generated predicted probabilities were used to classify patients as high or standard risk; the *high risk* group was defined as patients with a SORT generated risk of 30-day mortality of ≥ 5% and the *standard risk* group defined as those with a SORT generated risk of 30-day mortality of < 5%.

### Statistical analysis

Continuous data are reported as mean and standard deviation (SD) or median and interquartile range (IQR). An area under the receiver operator characteristic curve (AUROC) with 95% confidence intervals (CI) was drawn to assess the ability of SORT to predict in-hospital mortality. As the dataset was imbalanced in terms of a small number of in-hospital deaths, a precision-recall curve (PRC) was also drawn and the area under the PRC (AUPRC) calculated. A PRC reduces the impact of a large population of ‘true negative’ cases in a dataset with few events of interest (Saito & Rehmsmeier, 2015). The literature on calculating CI for a AUPRC is controversial (Boyd & Page, 2013); therefore, 95% CI is not reported for this metric.

## Results

In total, 3357 patients were identified. After excluding patients who were transferred to the NHS (*n* = 43, 1.3%) or to other independent hospitals (*n* = 9, 0.3%), the study population included 3305 patients. The mean age of patients was 51 years, the most frequent ASA grading was two (47.8%) and the majority of cases were elective (84.2%). The most common sites of surgery were the colon (812/3305, 24.6%), liver, pancreas and gallbladder (600/3305, 18.2%) and the rectum (376/3305, 11.4%, Table 1). In total there were 29 in-hospital deaths (0.88%). In comparison to patients who survived to discharge, patients who died were older, more likely to have cancer and other medical co-morbidities, had higher ASA scores and were more likely to be unplanned admissions to hospital.

**Table 1:** Study population demographics

	All patients ***N*** = 3305 ***N*** (%)	In-patient death ***N*** = 29 ***N*** (%)	Survived to discharge ***N*** = 3276 ***N*** (%)
**Mean age (SD)**	51.09 (15.88)	66.48 (9.38)	50.95 (15.86)
**Male gender (M:F)**	1592 (48.0)	18 (62.0)	1574 (48.0)
**ASA**			
**1**	1199 (36.3)	2 (6.9)	1197 (36.5)
**2**	1581 (47.8)	5 (17.2)	1576 (48.1)
**3**	477 (14.4)	13 (44.8)	464 (14.2)
**≥ 4**	48 (1.5)	9 (31.0)	39 (1.2)
**Elective admission**	2783 (84.2)	16 (55.2)	2767 (84.5)
**Type of surgery**			
**Oesophagus**	244 (7.38)	1 (3.5)	243 (7.42)
**Stomach**	310 (9.38)	0 (0.0)	310 (9.46)
**Duodenum**	34 (1.03)	0 (0.0)	34 (1.04)
**Small intestine**	189 (5.72)	2 (6.9)	187 (5.71)
**Large intestine**	812 (24.6)	4 (13.8)	808 (24.7)
**Rectum**	376 (11.4)	1 (3.45)	375 (11.4)
**HPB**	600 (18.2)	10 (34.5)	590 (18.0)
**Other**^a^	740 (22.4)	11 (37.9)	729 (22.3)
**Severity of procedure**			
**Intermediate**	286 (8.65)	0 (0.0)	286 (8.73)
**Major**	1374 (41.6)	12 (41.4)	1362 (41.6)
**Complex major/complex**	1645 (49.8)	17 (58.6)	1628 (49.7)
**Comorbidity**			
**Mean Charlson Index (IQR)**	0.692 (1)	3.59 (1)	0.667 (1)
**Cancer (yes/no)**	330 (10.0)	15 (52.0)	315 (9.6)

^a^Other: laparotomy, retroperitoneal surgery, adhesionolysis, aortic surgery, adrenalectomy

### The clinical performance of SORT

**Fig. 1:**
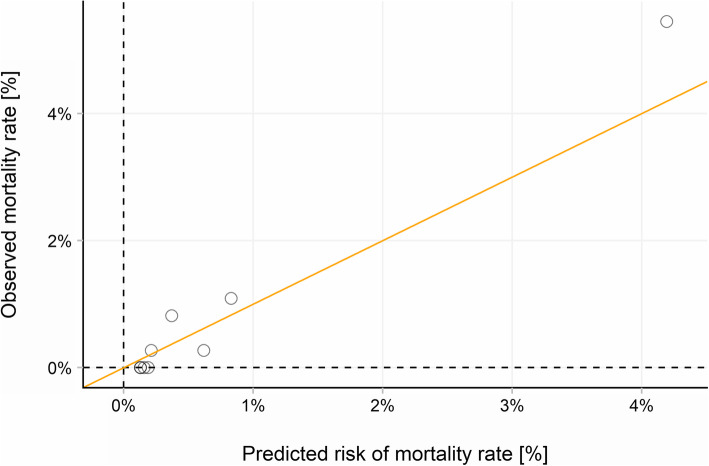
Observed versus predicted 30-day mortality at varying levels of risk

The observed and predicted mortality rates are shown in Table 2. For quantiles 1 to 4, the mean predicted mortality was < 0.2%, and there were no observed deaths in these groups. In quantiles 5 to 9, the mean predicted probability of death ranged from 0.21 to 4.19% (Fig. 1). Overall SORT under-predicted the number of deaths. Across the entire cohort of patients, 29 patients died. SORT predicted 25 of these. On an individual case level, the SORT predicted risk of in-hospital mortality ranged from 0.13 to 43.81%.

**Table 2:** Observed versus SORT predicted in-hospital mortality

Quantile	N patients	Mean SORT predicted probability of in-hospital mortality (%)	Observed in-hospital deaths ***N*** (%)	Predicted in-hospital deaths ***N***
**1**	368	0.129%	0 (0%)	0.47
**2**	367	0.129%	0 (0%)	0.47
**3**	367	0.156%	0 (0%)	0.57
**4**	367	0.188%	0 (0%)	0.69
**5**	368	0.213%	1 (0.03%)	0.78
**6**	367	0.370%	3 (0.09%)	1.36
**7**	367	0.617%	1 (0.03%)	2.27
**8**	367	0.829%	4 (0.12%)	3.04
**9**	367	4.193%	20 (0.61%)	15.39
**Total**	3305	0.758%	29 (0.88%)	25.1

**Fig. 2:**
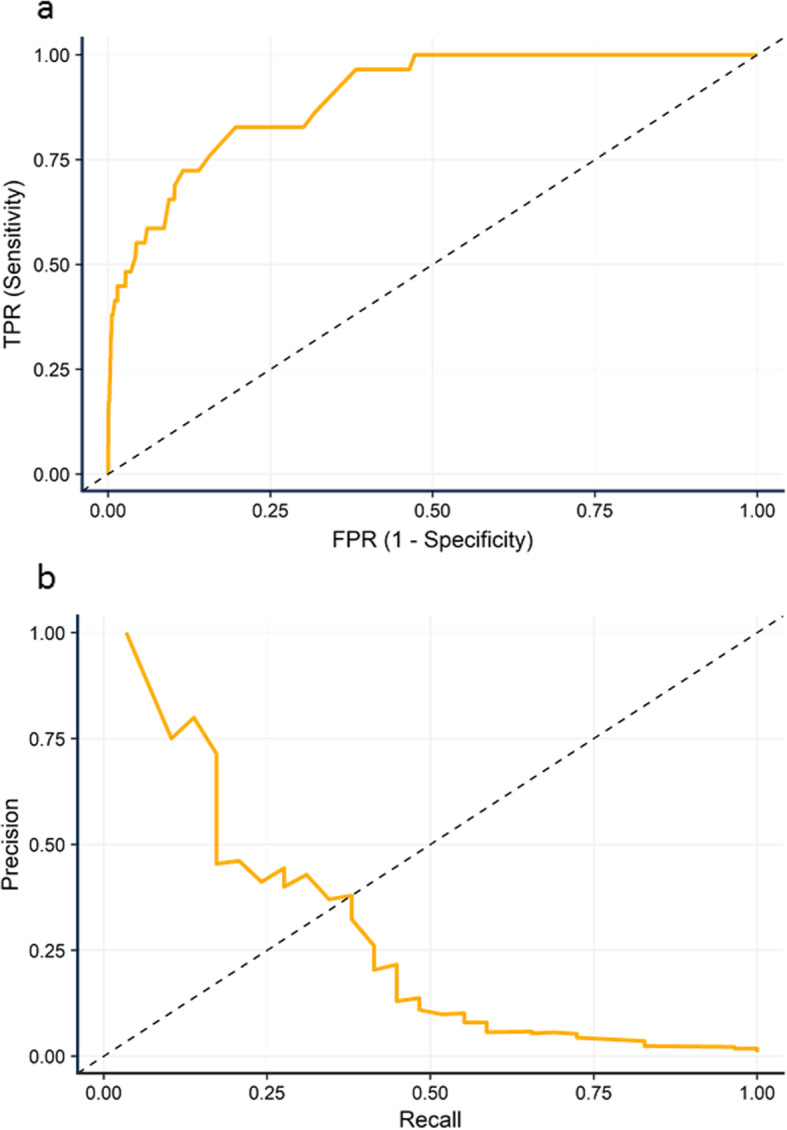
Predictive ability of SORT to discriminate patients at risk of in-hospital mortality. **a** AUROC for SORT for the prediction of in-hospital mortality. **b** Precision-recall curve for SORT for the prediction of in-hospital mortality

The AUROC c-statistic for SORT was 0.899 (95% CI 0.849 to 0.949, Fig. 2) suggesting good discriminative ability. However, the area under the precision-recall curve for SORT was 0.247 suggesting that the large proportion of true negatives may have artificially improved the ROC curve.

### The use of SORT to identify high-risk patients

Overall, 72/3305 (2.2%) patients had an individual predicted risk of post-operative in-hospital mortality of ≥ 5% and were therefore classified as *high risk* (Table 3). The remaining patients had SORT predicted mortalities of < 5% and were classified as *standard risk*. Patients in the high risk group were older, with higher Charlson Co-morbidity Indices and were more likely to have had emergency surgery in comparison to the standard risk group.

**Table 3:** Outcomes of patients stratified as ‘high risk’ by the Royal College of Surgeons recommendations

	Predicted risk of 30 day mortality ≥ 5% Total *n* = 72 *N* (%)	Predicted risk of 30 day mortality < 5% Total *n* = 3233 *N* (%)
Mean age (SD)	69.72 (15.83)	50.67 (15.63)
Mean Charlson Index (IQR)	2.24 (2)	0.658 (1)
Elective surgery	11 (15.3%)	2772 (85.7%)
Type of surgery		
Oesophagus	0 (0.00%)	244 (7.55%)
Stomach	2 (2.78%)	308 (9.53%)
Duodenum	2 (2.78%)	32 (1.0%)
Small intestine	4 (5.56%)	185 (5.72%)
Large intestine	19 (26.4%)	793 (24.5%)
Rectum	8 (11.1%)	368 (11.4%)
HPB	9 (12.5%)	591 (18.3%)
Other	28 (38.9%)^a^	712 (22.0%)
ICU admission		
Planned	36 (52.9%)	1180 (39.1%)
Unplanned	17 (25.0%)	101 (3.3%)
*Missing*	4	213
Median length of hospital stay (IQR)	18.34 (19.4)	5.27 (7.09)
In-hospital death	13 (18.1%)	16 (0.50%)
In-hospital death within 30 days	13 (18.1%)	8 (0.25%)

^a^‘Other’ surgery with a predicted risk of mortality of ≥ 5%: omentectomy, adhesionolysis, laparotomy (including for post-operative complications), excision of retroperitoneal tumour

In the high risk group, 57/72 (79%) patients were admitted to ICU post-operatively. Of these 57 ICU admissions, 17 were unplanned. In the standard risk group, 1494/3233 (46%) patients were admitted to ICU, 101 of whom were unplanned. There were more unplanned ICU admissions in the high risk group (17/68, 25.0%, missing data in four admissions) than the standard risk group (101/3020 (3.34%, missing data in 213 admissions). Within the high risk group, there were an additional 15 patients who were managed without post-operative ICU admission.

In the standard risk group, there were 16 deaths, although in comparison to patients stratified as high risk, significantly fewer patients died (16/3233, 0.49% versus 13/72, 18%, respectively). The median length of stay of high-risk patients was 18 days, in comparison to 5 days for the predicted standard-risk group.

## Discussion

In this large external validation study examining the performance of SORT in patients undergoing abdominal surgery, we found that SORT accurately predicted risk of post-operative death. It performed particularly well in low-risk patients, but under-predicted the risk of death in patients who were stratified as the highest risk. When SORT was used to identify patients at risk of adverse outcome, only 2.2% of the study population were identified as being high risk. In this high risk group, 25% patients had unplanned ICU admissions. If SORT had been used to identify patients at high risk of mortality and then to decide to electively admit to ICU, 25% of the observed unplanned ICU admission may have been avoided.

SORT was originally developed in 11,219 non-cardiac surgical patients (Protopapa et al., 2014) identified in the NCEPOD enquiry titled ‘Knowing the Risk’ (National Confidential Enquiry into Patient Outcomes and Death, 2011). Its authors successfully validated it in a separate population of 5569 non-cardiac patients, with an AUROC of 0.91 (Protopapa et al., 2014). There were 87 deaths by 30 days in this validation cohort, but SORT predicted only 73 (Protopapa et al., 2014). The present study shows a similar trend; there were 29 deaths, but SORT predicted 25.

Several further external validation studies have assessed the ability of SORT to predict 30-day mortality, although none in a mixed population of abdominal surgery patients. Wong et al. calculated SORT for 475 hepatectomies, reporting an AUROC of 0.82; however, SORT over-predicted the number of deaths, particularly in patients with the lowest risk profiles (Wong et al., 2017a). Oliver et al. assessed SORT in a mixed population of 1936 elective orthopaedic and general surgery procedures reporting an AUROC of 0.85 (Oliver, 2015). Like the present study, both of these studies reported low mortality rates (0.3% and 1.7%, respectively) and therefore contained a high proportion of true negatives, which may have led to an over-estimation of the performance of SORT. Marufu et al. assessed the performance of SORT in a population of hip fracture patients, who had a higher rate of death (5%). In this more balanced population, SORT did not perform as effectively, with an AUROC of only 0.70 (Marufu et al., 2016).

The predictive ability of risk stratification tools is frequently assessed using AUROCs and the *c*-statistic. However in populations where the outcome of interest is infrequent, such as the low mortality rate seen in the present study, AUROCs may over-estimate the performance of the model. This is due to impact of a large proportion of patients without the event (true negatives) in the calculation of specificity. In imbalanced populations, the more appropriate analysis may be the PRC, where true negatives do not feature in the calculation of precision (positive predicted value) or recall (sensitivity) (Saito & Rehmsmeier, 2015). The present study is the first to assess the performance of SORT using PRC as well as a ROC curve, finding that the performance of SORT was significantly poorer. This was notable in patients with the highest risk profiles, where SORT under-quantified their risk. In lower risk patients SORT performed well though. Arguably risk prediction tools in these patients are more useful than in patients with higher risk profiles, as the latter will have risk factors for poor outcome, such as advanced age, complex co-morbidity or emergency surgery which are readily identified by clinicians. A risk score that was able to generic patient-specific risk prediction would be useful in allowing focussed discussion between surgeons and their patients and improve informed consent, however.

Several other tools have been designed to predict post-operative morbidity and mortality, such as ASA (American Society of Anaesthesiologists, 2014) and the Portsmouth Physiological and Operative Severity Score for Mortality and Morbidity (P-POSSUM) (Prytherch et al., 1998). In an external validation study of 5569 patients, SORT was superior to ASA at predicting mortality, although both performed well (AUROCs of 0.91 and 0.87, respectively) (Protopapa et al., 2014). ASA is a population-based tool defining physical status not operative risk, and although widely used, misclassifications are common particularly amongst patients with multiple co-morbidities (Helkin et al., 2017). The performance of SORT is yet to be compared to that of P-POSSUM. A limitation of P-POSSUM is that it requires laboratory data, a chest radiograph and electrocardiogram, making it more difficult to calculate than SORT.

Once risk prediction is established as being accurate, the next question is regarding the discrete level of risk that qualifies a patient as ‘high risk’. The RCS recommend using a predicted risk of death of ≥ 5% to identify high-risk patients (Royal College of Surgeons of England, 2018). This represents a departure from previous guidance that categorised patients as high risk if they had a predicted risk of death of ≥ 10% (Royal College of Surgeons of England, 2011). The present study is the first to assess ICU utilisation following the new recommendation of a threshold of 5%, and the first to use SORT to stratify patients. We demonstrate that lowering the threshold to 5% does not generate large volumes of new post-operative ICU admissions; only 2.2% of the study population met the criteria for direct ICU admission, and most of these had already been recognised as requiring post-operative ICU care. This group of additional ICU admissions represents only 0.45% of the study population. Of note, 25% of the high-risk group had unplanned ICU admissions. These patients represent a sub-group of high-risk patients that could have been identified pre-operatively by SORT and electively admitted to ICU. However, there were also patients in the high risk group who were managed without ICU admission, and conversely patients in the standard risk group that had unplanned ICU admissions or died in hospital. In the standard risk group, there were 16 deaths, suggesting that using a predicted mortality of 5% may yet be too high to safely identify all patients at risk of death.

Historically post-operative ICU admission has been thought to be of benefit as it permits rapid recognition and treatment of life-threatening post-operative complications. A study of 572,598 general surgical procedures found that a patient who receives post-operative ward-based care but then requires unplanned ICU admission has twice the risk of 30-day mortality (Gillies et al., 2017). In elective surgery, a recent study of 44,814 patients found no association between direct admission to ICU following surgery and in-hospital mortality however (Kahan et al., 2017). These findings may be explained by advances in surgical and anaesthetic techniques that have reduced the physiological disturbance caused by surgery and therefore reduced the impact of ICU-based care. In the present study, half of the patients in the standard risk group were admitted to ICU post-operatively. Given the acuity of the surgical procedures, this is not an unexpected finding, but in the future a proportion of these patients may be eligible to receive critical care interventions, such as telemetry or vasopressors, outside of the traditional ICU.

Within the standard risk group, 3.3% of patients had an unplanned ICU admission. These patients would not have been identified if risk stratification was restricted to SORT and the 5% mortality threshold. It is therefore important to highlight that risk tools serve to aid, as opposed to replace clinical judgement. None of the previously described scores have been directly compared to clinical opinion, but when assessing pre-operative risk, guidelines recommend that risk tools are used in conjunction with surgical judgement (Royal College of Surgeons of England, 2018). In keeping with this, the American College of Surgeons National Surgical Quality Improvement Programme risk tool has an in-built option to allow surgeons to modify risk calculations if they deem necessary (Bilimoria et al., 2013).

This study uses data collected from patients treated in five independent hospitals in the UK, a sector of healthcare that is traditionally thought to deliver simple treatments to stable patients. When comparing the demographics of this population to that of a contemporaneous NHS population of 16,788 surgical patients (Protopapa et al., 2014), there are important similarities. High ASA classifications were common (ASA 3 and 4 were found in 19.9% and 2.7%, respectively, in the NHS study (Protopapa et al., 2014), and 14.4% and 1.5%, respectively, in the present study) and the majority of patients were undergoing major or complex-major operations (32.7% and 34.2% patients, respectively in the NHS study (Protopapa et al., 2014) and 41.6% and 49.8%, respectively, in the present study). The mortality rate was also similar (1.8% in the NHS study and 0.88% in the present study) and comparable to reported rates of 1.1 to 1.9% in other large NHS-based population studies of surgical patients (Abbott et al., 2017; Pearse et al., 2006).

There are some important limitations to the present study. SORT was initially developed to predict 30-day mortality, but the present study was limited to in-hospital death as we were unable to collect data on patient outcomes after discharge. We also unable to capture the outcomes of patents who were transferred to the NHS or other healthcare providers. However, these cases represented only 1.6% of the study population. In some cases, we were unable to determine the rationale for post-operative ICU admission so these cases were excluded from this sub-analysis. In the remaining cases, we assumed that ICU admissions categorised as unplanned were categorised using clinical need. However, a proportion of these may represent elective admissions where the operating surgeon has failed to book a bed, and were not truly unplanned admissions. It was not possible to sub-classify procedure urgency beyond elective or unplanned, so we were unable to identify which patients were truly ‘expedited’ or ‘emergency’ procedures. This may mean that true ‘emergency procedures’ are under-represented in the study population, leading to under-estimation of ICU capacity needed to implement the 5% risk threshold. It may also mean the performance of SORT described in the present study is not as good as could be if all variations of procedure urgency were included. We are unable to directly compare rates of total ICU admission in this study to that of other intuitions as we can find no contemporary multicentre studies conducted in a UK population that report this outcome. Notably, all patients in the present study underwent major abdominal surgery (patients undergoing minor or intermediate procedures were excluded).

In summary, this large study externally validates SORT in a population of patients undergoing major abdominal surgery. SORT performed particularly well in patients with low-risk profiles, but under-predicted the number of deaths in patients with the highest risk, which is a significant limitation in this subgroup. When SORT was used to identify patients with a predicted post-surgery mortality of ≥ 5% and therefore requiring direct ICU admission some patients who were stratified as standard risk ultimately required unplanned ICU admission. However, SORT did identify high-risk patients who had unplanned ICU admissions, demonstrating the value of using SORT in conjunction with clinical judgement.

## Supplementary Information

**Additional file 1. Appendix**

## Data Availability

The datasets generated and/or analysed during the current study are not publicly available as they contain commercially sensitive information but are available from the corresponding author on reasonable request.
