# Supplementary material for: External validation of the Surgical Outcome Risk Tool (SORT) in 3305 abdominal surgery patients in the independent sector in the UK
Source: Perioper Med (Lond). 2021 Jan 26;10:4. doi: 10.1186/s13741-020-00173-1 (PMC7836595; doi:10.1186/s13741-020-00173-1)
Supplement: Supplementary file 1 — Additional file 1. Appendix [file 13741_2020_173_MOESM1_ESM.docx]

**Appendix 1 - eligible procedure codes**

**Stomach**

A2720 Proximal gastric vagotomy

A2730 Highly selective vagotomy

A2780 Vagotomy with pyloroplasty

G2710 Total gastrectomy and excision of surrounding tissue

G2800 Partial gastrectomy

G2810 Partial gastrectomy and excision of surrounding tissue

G3070 X Sleeve gastrectomy (including laparoscopic)

G3080 X Laparoscopic gastric banding

G3090 X Gastric bypass eg. Roux en Y for morbid obesity (including laparoscopic)

G3100 Laparoscopic biliary gastric bypass

G3210 Gastro-jejunostomy

G3220 Revision of gastro-jejunostomy

G3520 Closure of perforated ulcer of stomach

G3580 Laparoscopic closure of peptic ulcer

G3610 Gastropexy (for reflux)

G3800 Open operations on stomach not elsewhere classified

G4010 Pyloromyotomy

G4030 Pyloroplasty

**Duodenum**

G5000 Open excision of lesion of duodenum

G5010 Open excision of congenital lesion of duodenum (including malrotation)

G5020 Robotic laparoscopic switch of duodenum with vertical gastrectomy and biliary pancreatic diversion

G5100 Bypass of duodenum

G5320 Closure of perforated ulcer of duodenum

G6082 Open resection of small intestine tumour

**Small intestine**

G5810 Excision of jejunum

G6000 Open formation of jejunostomy

G6080 Laparoscopically assisted resection of small intestine

G6100 Bypass of jejunum

G7100 Bypass of ileum

G7250 Ileoanal anastomosis and creation of pouch

G7402 Open formation of ileostomy

G7403 Laparoscopic ileostomy

G7512 Revision of ileostomy - local

G7513 Revision of ileostomy - laparotomy

G7530 Closure of ileostomy (as sole procedure)

**Large intestine**

H0210 Appendicectomy

H0280 Laparoscopic appendicectomy

H0310 Drainage of abscess of appendix or drainage of intra-abdominal abscess

H0510 Total excision of colon and ileorectal anastomosis

H0610 Extended excision of right hemicolon

H0700 Right hemicolectomy

H0750 Laparoscopically assisted right hemicolectomy

H0800 Excision of transverse colon

H0900 Excision of left hemicolon

H1000 Excision of sigmoid colon

H1200 Excision of lesion of colon (transabdominal)

H1300 Bypass of colon

H1542 Closure of colostomy

H1581 Laparoscopic colostomy and stoma formation (including revision)

H1590 Open formation of colostomy

H1700 Intra abdominal manipulation of colon for intussusception (as sole procedure)

H1880 Laparoscopically assisted left colon resection

**Rectum**

G7250 Ileoanal anastomosis and creation of pouch

H0410 Panproctocolectomy and ileostomy

H0480 Abdominal revision of restorative proctocolectomy

H3310 Abdominoperineal pull through resection with colo-anal anastomosis +/- colonic pouch and associated stoma

H3320 Abdominoperineal resection of rectum and anus

H3322 Laparoscopic abdominoperineal resection

H3332 Anterior resection - high (i.e. colorectal anastomosis above the peritoneal reflection)

H3334 Anterior resection - low (i.e. colorectal anastomosis at or below the peritoneal reflection)

H3362 Hartmann's procedure

H3363 Colectomy and colostomy and preservation of rectum

H3364 Laparoscopic anterior resection - high (i.e. colorectal anastomosis above the peritoneal reflection)

H3365 Laparoscopic anterior resection - low (i.e. colorectal anastomosis at or below the peritoneal reflection)

H3380 Partial excision of rectum and sigmoid colon for prolapse

H3381 Total Mesorectal Excision (TME)

H3382 Proctectomy

H3390 Reversal of Hartmann's procedure

H3500 Fixation of rectum for prolapse

H3580 Laparoscopic rectopexy

H3581 Robotic assisted laparoscopic rectopexy

**Major vessels**

L7980 Repair of wound of major artery or vein of abdomen (including aorta and vena cava)

**Oesophagus**

A2781 Laparoscopic vagotomy/seromyotomy

G0100 Oesophagectomy/Oesophagogastrectomy with anastomosis in chest

G0220 Total oesophagectomy and interposition of intestine

G0260 Endoscopically assisted oesophagectomy

G0300 Sub-total oesophagectomy with anastomosis in neck

G0400 Open excision of lesion of oesophagus

G0500 Bypass of oesophagus

G0740 Repair of ruptured oesophagus

G0920 Oesophagocardiomyotomy (Heller's operation)

G0980 Thorascopic oesophagogastric myotomy

G1400 VATS excision lesion of oesophagus

G2312 Transthoracic repair of paraoesophageal hiatus hernia

G2320 Transthoracic repair of diaphragmatic hernia (acquired)

G2330 Transabdominal repair of hiatus hernia

G2331 Laparoscopic repair of hiatus hernia with anti-reflux procedure (e.g. fundoplication)

G2340 Transabdominal repair of diaphragmatic hernia

G2400 Transthoracic fundoplication & gastroplasty

G2402 Transthoracic fundoplication

G2430 Transabdominal anti-reflux operations

G2590 Revision of anti-reflux operations

**Other abdominal organs (mainly digestive)**

B2222 Adrenalectomy - bilateral (open)

B2223 Adrenalectomy - bilateral (laparoscopic)

B2224 Robotic assisted laparoscopic adrenalectomy - bilateral

B2232 Adrenalectomy - unilateral (open)

B2233 Adrenalectomy - unilateral (laparoscopic)

B2234 Robotic assisted laparoscopic adrenalectomy

**Other abdominal organs (mainly digestive – HPB)**

J0200 Partial hepatectomy (left hepatectomy or resection of up to three segments) +/- cholecystectomy

J0210 Hemihepatectomy (resection of four or more segments) +/- cholecystectomy

J0220 Robotic assisted hemihepatectomy

J0310 Resection of liver tumour(s)

J0400 Repair of liver (including therapeutic laparoscopic operations on liver)

J0510 Open drainage of liver

J0740 Open hepatectomy and ablation

J0780 Radiofrequency thermocoagulation of liver with scalpel liver resection

J1800 Cholecystectomy (including mini-cholecystectomy)

J1820 Cholecystectomy with exploration of common bile duct

J2720 Partial excision of bile duct and anastomosis of bile duct to duodenum/jejunum

J2800 Excision of lesion of bile duct

J2900 Anastomosis of hepatic duct

J3000 Anastomosis of common bile duct

J3200 Repair of bile duct

J3300 Incision of bile duct (including exploration for calculus removal)

J3500 Sphincterotomy of bile duct and pancreatic duct using duodenal approach

J5480 Pancreatic transplant (including sequential pancreatic transplant)

J5481 Pancreatic with kidney transplant (simultaneous pancreas SPK)

J5500 Total pancreatectomy and excision of surrounding tissue

J5520 Total pancreatectomy

J5610 Pancreatoduodenectomy and excision of surrounding tissue (Whipple's Procedure)

J5700 Distal pancreatectomy

J5711 Pancreatectomy with autologous islet cell transplantation

J5712 Robotic assisted distal pancreatectomy

J5750 Laparoscopic distal pancreatectomy

J5800 Excision of lesion of pancreas

J5900 Anastomosis of pancreatic duct (to another viscus)

J6100 Open drainage of lesion of pancreas

J6180 Drainage of pancreatic abscess

J6200 Incision of pancreas

J6300 Open examination of pancreas

J6600 Therapeutic percutaneous operations on pancreas

J6900 Open splenectomy

J6980 Laparoscopic splenectomy

**Peritoneum (General)**

T3010 Laparotomy for post-operative haemorrhage

T3080 Laparotomy and repair of multiple visceral trauma

T3410 Open drainage of subphrenic abscess

T3600 Wedge excision or removal of omentum (as sole procedure)

T3910 Excision of retroperitoneal tumour, +/-ureterolysis

T3920 Multivisceral resection of retroperitoneal sarcoma

T3930 Retroperitoneal abscess

T3980 Excision of presacral tumour

T3990 Excision of retroperitoneal neuro-endocrine lesion

T4130 Freeing of adhesions of peritoneum

T4300 Laparoscopic adhesiolysis (including biopsy)

T4302 Open adhesiolysis (including biopsy)
